# Supplementary material for: Spatial targeting of Screening + Eave tubes (SET), a house-based malaria control intervention, in Côte d’Ivoire: A geostatistical modelling study
Source: PLOS Glob Public Health. 2021 Nov 15;1(11):e0000030. doi: 10.1371/journal.pgph.0000030 (PMC10021308; doi:10.1371/journal.pgph.0000030)
Supplement: S1 File — (DOCX) [file pgph.0000030.s001.docx]

Supporting Information

**S1 Table. Classification of material appropriateness.**

# S1 Table

| **Appropriateness** | **Material** |
| --- | --- |
| Wall appropriate | Cement, Bricks, Cement blocks, Stone with lime / Cement |
| Wall inappropriate | Dirt, Bamboo with mud, Uncovered adobe, Covered adobe, Stone with mud, Cane / Palm / Trunks, Wood planks / Shingles, Reused wood, Other, Plywood, No walls |
| Roof appropriate | Metal, Cement, Ceramic tiles, Roofing shingles |
| Roof inappropriate | Thatch / Palm leaf, Palm / Bamboo, Other, Wood planks, Wood, Sod, No roof, Cardboard, Rustic mat |

**S1 Table. Classification of material appropriateness.** Source: Demographic Health Survey 2011, Côte d'Ivoire [1].

# References

1. ICF International. Côte d’Ivoire Enquéte Démographique et de Santé et Indicateurs Multiples 2011-2012 [Internet]. 2013;Available from: http://dhsprogram.com/pubs/pdf/FR272/FR272.pdf
