# Supplementary material for: Spatial targeting of Screening + Eave tubes (SET), a house-based malaria control intervention, in Côte d’Ivoire: A geostatistical modelling study
Source: PLOS Glob Public Health. 2021 Nov 15;1(11):e0000030. doi: 10.1371/journal.pgph.0000030 (PMC10021308; doi:10.1371/journal.pgph.0000030)
Supplement: S2 File — (DOCX) [file pgph.0000030.s002.docx]

Supporting Information

**S2 Table: Comparation of land use classification between DHS and Grace et al. (2019) methodology.**

**S2 Fig: Location of Côte d’Ivoire DHS clusters corrected with Grace et al. methodology.**

# S2 Table and S2 Fig

Rural localization adjustment

To the DHS survey coordinates we applied the methodology of Grace et al.^1^ with the aim to correct the rural misplacement generated by their privacy policy. This methodology does not aim to identify the survey respondents but to provide a more realistic cluster location. This is useful when using a buffer to extract covariates. Applying this method, we reduced the size of the rural buffer from 10km (recommended by the DHS methodology) to 2km.

To check whether the methodology provided more accurate results, we compared the new to the old location. For each rural cluster (n = 181) we extracted the values of degree of urbanization;^2^ urban, rural and no population. We classified the cluster based on the most common value inside the buffer and compared to the DHS classification of urban or rural.

To compensate the higher probability of no population inside the 10km DHS buffer, we created a 2km buffer around the old DHS clusters to compare the results.

|  | **Land use category** | |  | | |
| --- | --- | --- | --- | --- | --- |
|  | Rural | Urban | No population | Total clusters | Correct classification |
| N° of new rural clusters  2km buffer | 54 | 6 | 121 | 181 | **30%** |
| N° of old rural clusters  2km buffer | 45 | 6 | 130 | 181 | **25%** |
| N° of old rural clusters  10km buffer | 33 | 0 | 148 | 181 | **18%** |

**S2 Table: Comparation of land use classification between DHS and Grace et al. (2019) methodology.**

We can observe that although the new location misclassifies six clusters into urban category, it does a better job (12% improvement) to identify rural areas than the DHS original locations. Many clusters are classified as having no population because villages or rural towns are small, and in this case most pixels within a buffer are unpopulated.

This map displays the location of urban and rural cluster after applying the methodology:

**
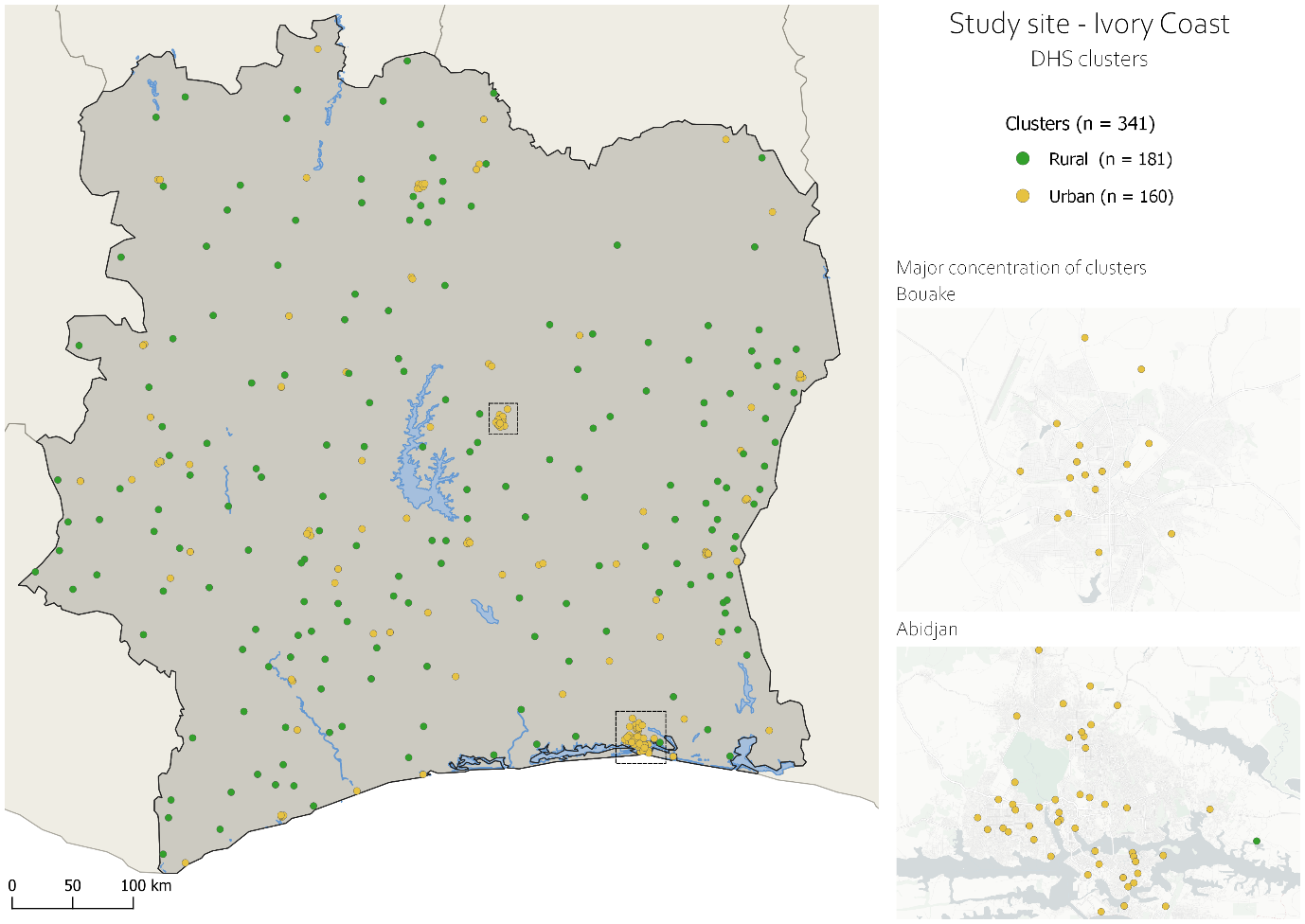
**

**S2 Fig: Location of Côte d’Ivoire DHS clusters corrected with Grace et al. methodology.**

# References

1 Grace K, Nagle NN, Burgert-Brucker CR, *et al.* Integrating Environmental Context into DHS Analysis While Protecting Participant Confidentiality: A New Remote Sensing Method. *Popul Dev Rev* 2019; **45**: 197–218.

2 Pesaresi M, Freire S. GHS-SMOD R2016A - GHS settlement grid, following the REGIO model 2014 in application to GHSL Landsat and CIESIN GPW v4-multitemporal (1975-1990-2000-2015). *Eur Comm Jt Res Cent* 2016. http://data.europa.eu/89h/jrc-ghsl-ghs_smod_pop_globe_r2016a.
