# Supplementary material for: Spatial targeting of Screening + Eave tubes (SET), a house-based malaria control intervention, in Côte d’Ivoire: A geostatistical modelling study
Source: PLOS Glob Public Health. 2021 Nov 15;1(11):e0000030. doi: 10.1371/journal.pgph.0000030 (PMC10021308; doi:10.1371/journal.pgph.0000030)
Supplement: S3 File — (DOCX) [file pgph.0000030.s003.docx]

Supporting Information

**S3 Table: Description of covariates used in the geostatistical model and priority classification.**

**S3 Fig. Maps of the covariates used in the paper.**

# S3 Table and S3 Fig

Covariate data sources

Table

| **Variable** | **Reference** | **Covariate proxy** | **Spatial resolution** | **Year of dataset** | **Source** |
| --- | --- | --- | --- | --- | --- |
| Food and non-food production crops | Institute IFPR. 2019 ^1^ | Economic development | ~ 10km | 2010 | <https://doi.org/10.7910/DVN/PRFF8V/KYZD6E> |
| Travel time to nearest city | Weiss et al. 2018 ^2^ | Access to services and economic capacity | ~1km | 2015 | <https://figshare.com/articles/Travel_time_to_cities_and_ports_in_the_year_2015/7638134/3> |
| Urbanisation | Pasaresi M, Freire S. 2016 ^3^ | Degree of urbanisation | ~1km | 2015 | <https://ghsl.jrc.ec.europa.eu/ghs_smod2019.php> |
| Aridity | Abatzoglou et al. 2018 ^4^ | Agricultural development | ~5km | 2019 | <http://www.climatologylab.org/> |
| Plasmodium falciparum parasite rate | Weiss DJ, Lucas TCD, Nguyen M, et al. 2019 ^5^ | Malaria transmission | ~5km | 2019 | <https://malariaatlas.org/> |
| Population | Worldpop, Southampton, UK ^6^ | Population | ~100m | 2020 | <https://www.worldpop.org/geodata/summary?id=49693> |

**S3 Table: Description of covariates used in the geostatistical model and priority classification.**

Fig
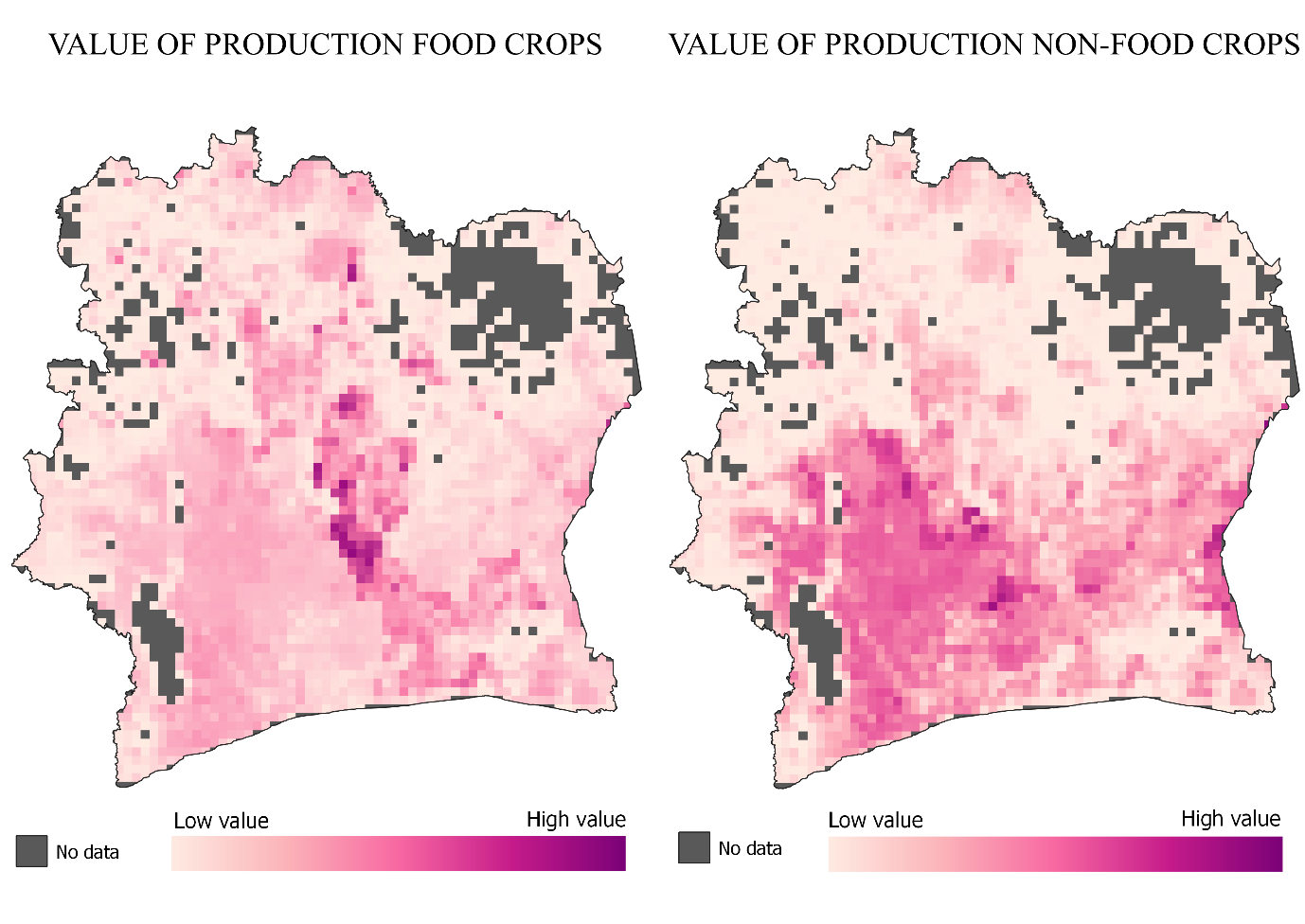

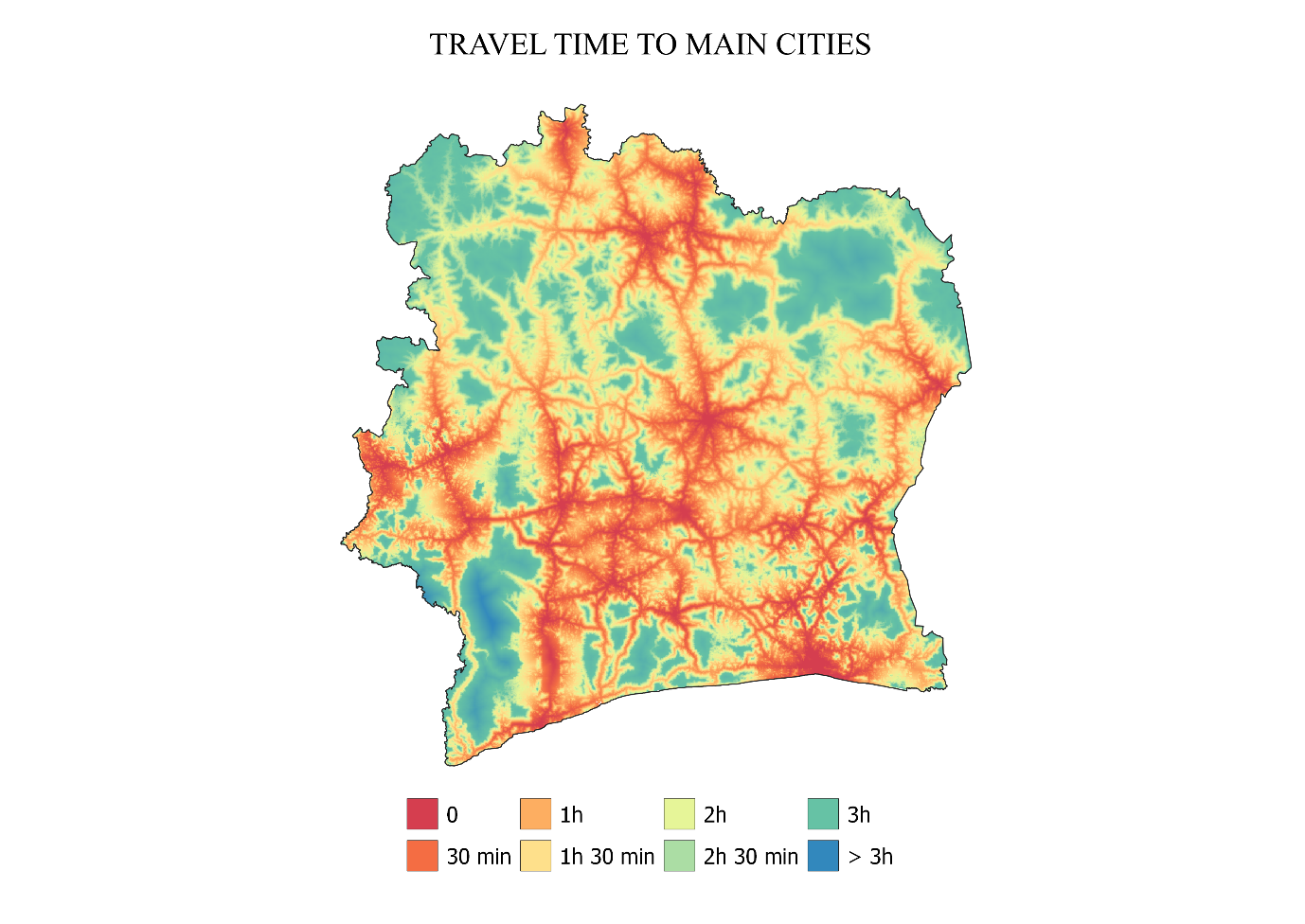

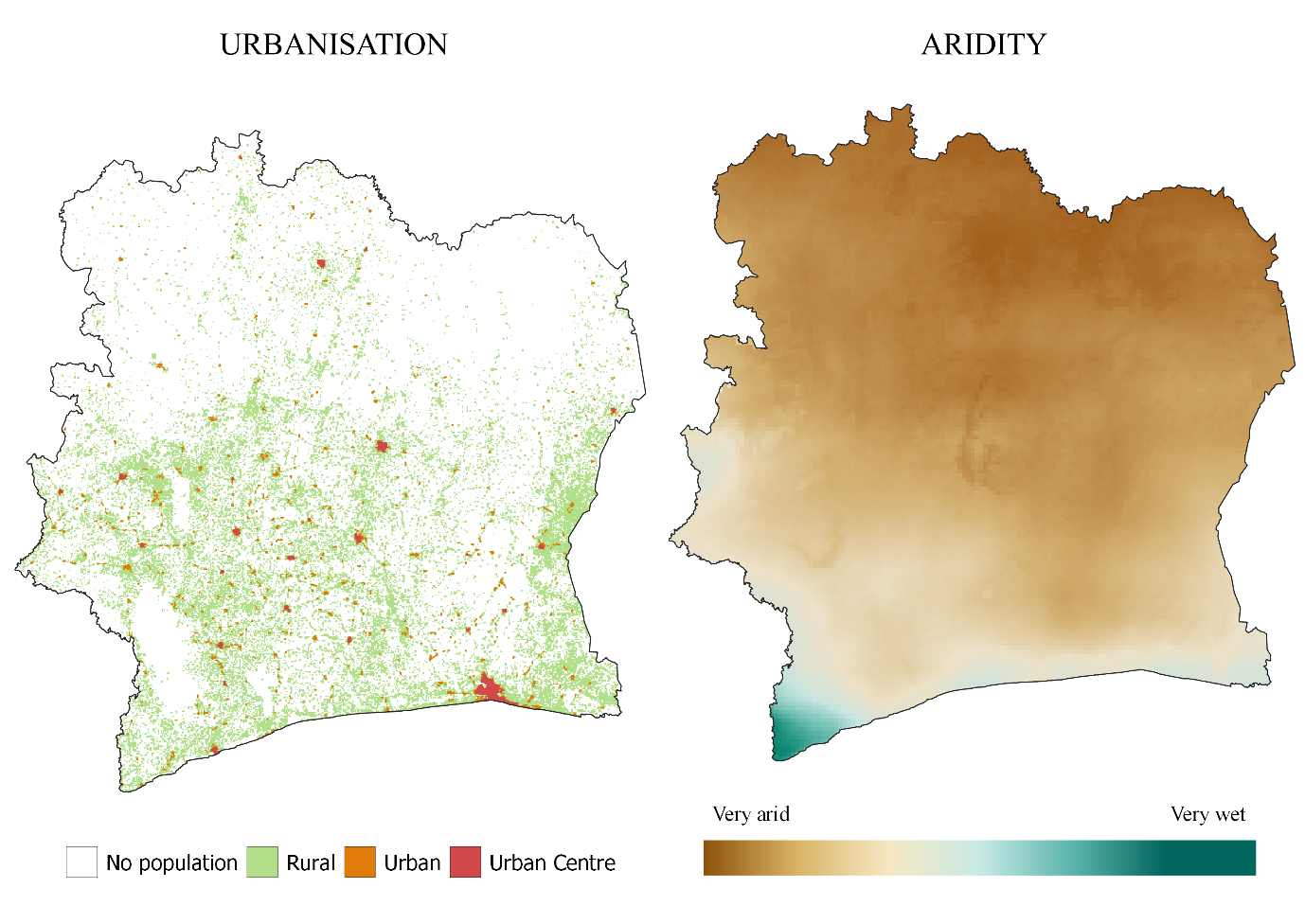


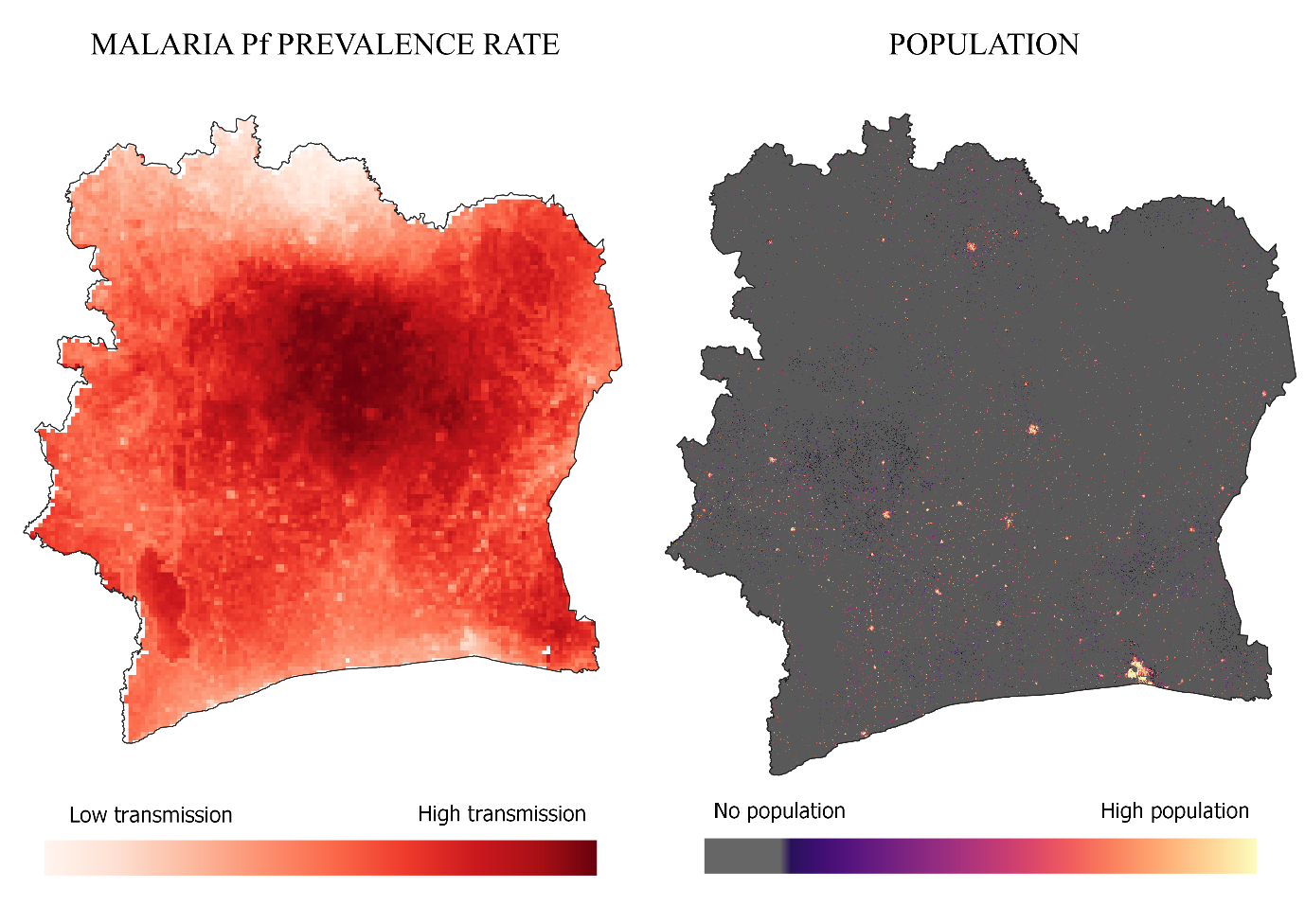


**S3 Fig. Maps of the covariates used in the paper.**

# References

1 Institute IFPR, Agriculture CP for BD in, CGIAR Research Program on Policies and Markets I, *et al.* spam2010v2r0_global_val_prod_agg.geotiff.zip. Glob. Spat. Crop Prod. Stat. Data 2010 Version 2.0. 2019. DOI:doi/10.7910/DVN/PRFF8V/KYZD6E.

2 Weiss DJ, Nelson A, Gibson HS, *et al.* A global map of travel time to cities to assess inequalities in accessibility in 2015. *Nature* 2018; **553**: 333–6.

3 Pesaresi M, Freire S. GHS-SMOD R2016A - GHS settlement grid, following the REGIO model 2014 in application to GHSL Landsat and CIESIN GPW v4-multitemporal (1975-1990-2000-2015). *Eur Comm Jt Res Cent* 2016. http://data.europa.eu/89h/jrc-ghsl-ghs_smod_pop_globe_r2016a.

4 Abatzoglou JT, Dobrowski SZ, Parks SA, Hegewisch KC. TerraClimate, a high-resolution global dataset of monthly climate and climatic water balance from 1958-2015. *Sci Data* 2018; **5**: 1–12.

5 Weiss DJ, Lucas TCD, Nguyen M, *et al.* Mapping the global prevalence, incidence, and mortality of Plasmodium falciparum, 2000–17: a spatial and temporal modelling study. *Lancet* 2019; **394**: 322–31.

6 Bondarenko M, Kerr D, Sorichetta A, Tatem AJ. Census/projection-disaggregated gridded population datasets, adjusted to match the corresponding UNPD 2020 estimates, for 51 countries across sub-Saharan Africa using building footprints. UK, 2020 https://www.worldpop.org/geodata/summary?id=49693.
