# Supplementary material for: Spatial targeting of Screening + Eave tubes (SET), a house-based malaria control intervention, in Côte d’Ivoire: A geostatistical modelling study
Source: PLOS Glob Public Health. 2021 Nov 15;1(11):e0000030. doi: 10.1371/journal.pgph.0000030 (PMC10021308; doi:10.1371/journal.pgph.0000030)
Supplement: S4 File — (DOCX) [file pgph.0000030.s004.docx]

Supporting Information

**S4 Fig. Wall and roof materials per urban and rural clusters**

# S4 Fig

Wall and roof materials used in Côte d’Ivoire.


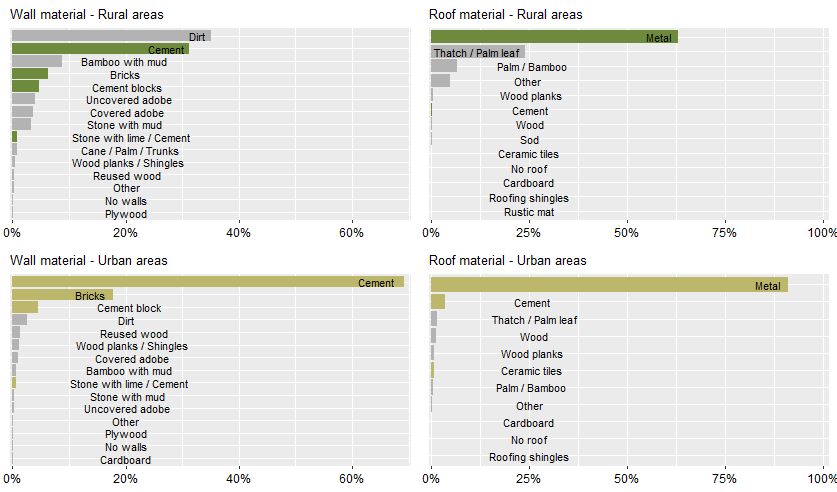


**S4 Fig. Wall and roof materials per urban and rural clusters**. Green (in rural areas) or beige (in urban areas) bars represent those materials which are suitable for implementation of SET. Source: Source: Demographic Health Survey 2011, Côte d'Ivoire ^1^.

# References

1 ICF International. Côte d’Ivoire Enquéte Démographique et de Santé et Indicateurs Multiples 2011-2012. 2013. http://dhsprogram.com/pubs/pdf/FR272/FR272.pdf.
