# Supplementary material for: Spatial targeting of Screening + Eave tubes (SET), a house-based malaria control intervention, in Côte d’Ivoire: A geostatistical modelling study
Source: PLOS Glob Public Health. 2021 Nov 15;1(11):e0000030. doi: 10.1371/journal.pgph.0000030 (PMC10021308; doi:10.1371/journal.pgph.0000030)
Supplement: S5 File — (DOCX) [file pgph.0000030.s005.docx]

Supporting Information

**S5 Fig. Estimates (a) and standard error (b) of suitability generated from the geospatial model.**

# S5 Fig

Estimates and standard error of the geospatial model.


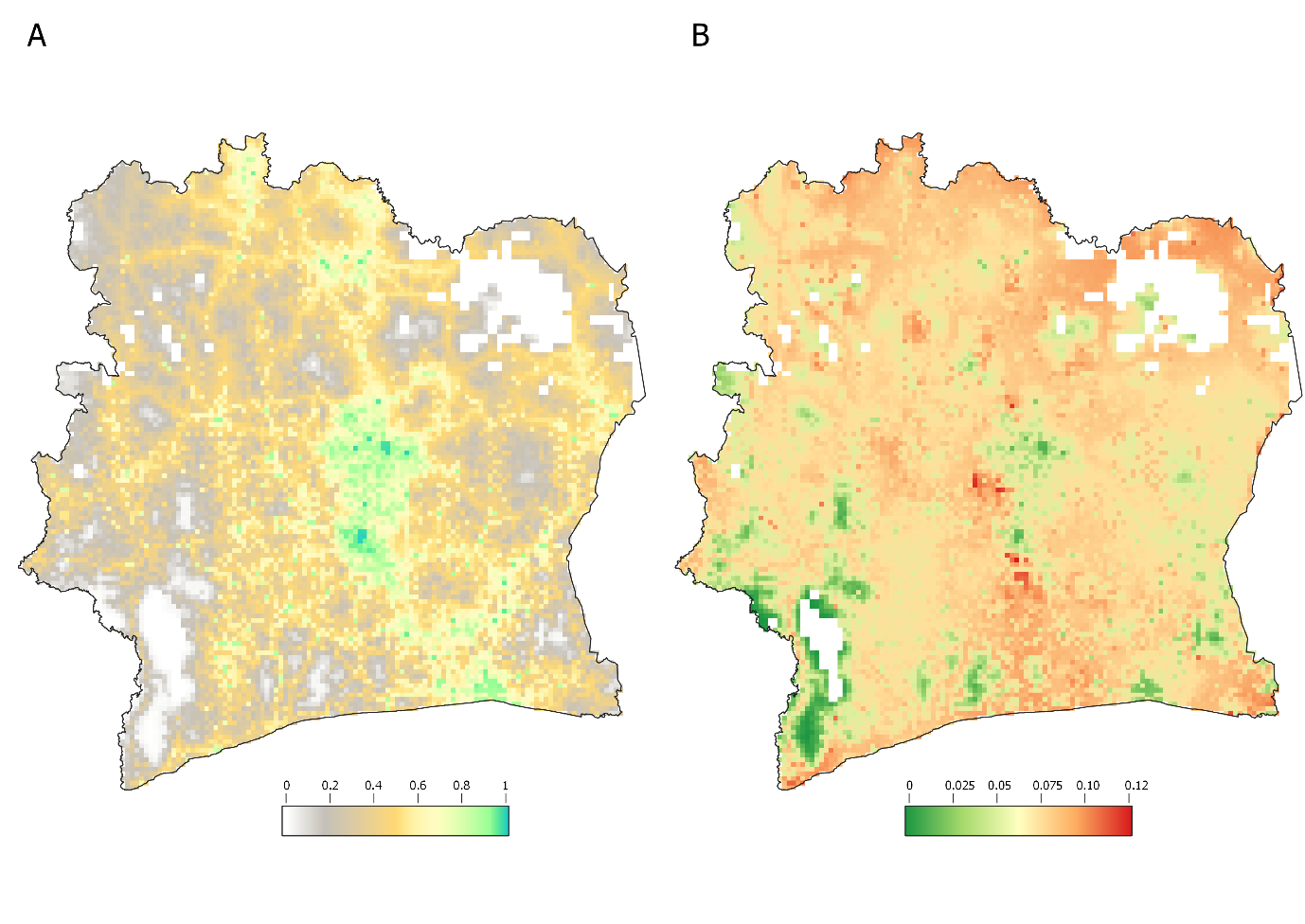


**S5 Fig. Estimates (a) and standard error (b) of suitability generated from the geospatial model.**
