# Supplementary material for: Spatial targeting of Screening + Eave tubes (SET), a house-based malaria control intervention, in Côte d’Ivoire: A geostatistical modelling study
Source: PLOS Glob Public Health. 2021 Nov 15;1(11):e0000030. doi: 10.1371/journal.pgph.0000030 (PMC10021308; doi:10.1371/journal.pgph.0000030)
Supplement: S6 File — (DOCX) [file pgph.0000030.s006.docx]

Supporting Information

**S6 Figure. Sensitivity analysis with an improvement of 20% on the household’s suitability for SET**

**S6 Table. Full list of priority departments.**

# S6 Fig

**Sensitivity analysis with an improvement of 20% on the household’s suitability for SET. A) Geostatistical output. B) Priority areas for ground-truthing.**


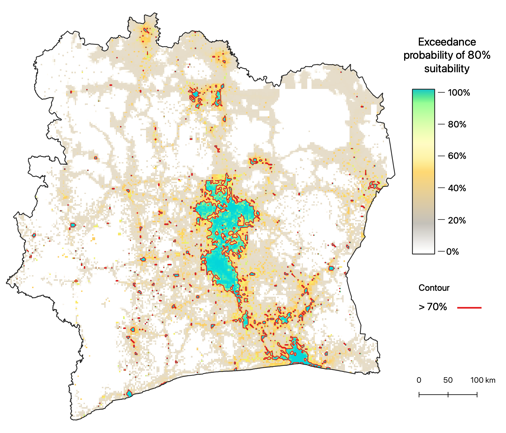

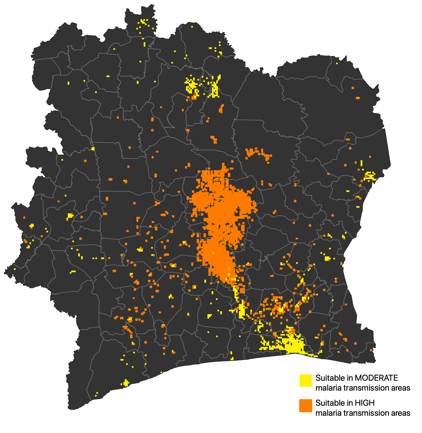


B

A

# S6 Table

* Z-score accessibility was changed the sign as smaller values are more priority than higher values

|  |  | COVARIATES WITHIN THE DEPARTMENTS | | | | Z-SCORE | | | | |
| --- | --- | --- | --- | --- | --- | --- | --- | --- | --- | --- |
| **Rank** | **Department** | **Suitable area (m2)** | **Mean time (min) to the nearest city** | **Mean malaria incidence** | **Mean exceendance probability** | **z-score accessibility** | **z-score malaria** | **z-score exceedance** | **z-score area** | **total*** |
| 1 | Bouaké | 997667040 | 16.638 | 0.698 | 0.946 | -0.889 | 1.997 | 0.700 | 3.703 | 7.290 |
| 2 | Botro | 598600224 | 34.534 | 0.770 | 0.910 | -0.186 | 2.474 | 0.244 | 1.959 | 4.863 |
| 3 | Yamoussoukro | 872958660 | 20.128 | 0.462 | 0.924 | -0.752 | 0.436 | 0.419 | 3.158 | 4.765 |
| 4 | Sakassou | 673425252 | 41.809 | 0.731 | 0.883 | 0.101 | 2.213 | -0.116 | 2.286 | 4.282 |
| 5 | Abidjan | 623541900 | 2.400 | 0.209 | 0.992 | -1.449 | -1.240 | 1.297 | 2.068 | 3.574 |
| 6 | Béoumi | 448950168 | 44.532 | 0.740 | 0.898 | 0.208 | 2.271 | 0.086 | 1.305 | 3.454 |
| 7 | Tiébissou | 673425252 | 43.375 | 0.593 | 0.829 | 0.162 | 1.298 | -0.804 | 2.286 | 2.618 |
| 8 | Attiégouakro | 424008492 | 34.383 | 0.484 | 0.880 | -0.191 | 0.580 | -0.153 | 1.196 | 1.814 |
| 9 | Toumodi | 648483576 | 42.144 | 0.404 | 0.859 | 0.114 | 0.050 | -0.421 | 2.177 | 1.692 |
| 10 | Katiola | 99766704 | 52.607 | 0.784 | 0.880 | 0.525 | 2.561 | -0.142 | -0.222 | 1.672 |
| 11 | Adzopé | 49883352 | 10.817 | 0.321 | 0.999 | -1.118 | -0.499 | 1.387 | -0.440 | 1.566 |
| 12 | Didievi | 224475084 | 45.090 | 0.614 | 0.887 | 0.230 | 1.438 | -0.055 | 0.323 | 1.477 |
| 13 | Akoupé | 74825028 | 24.130 | 0.333 | 0.993 | -0.595 | -0.422 | 1.312 | -0.331 | 1.154 |
| 14 | Korhogo | 49883352 | 2.083 | 0.205 | 0.999 | -1.462 | -1.265 | 1.384 | -0.440 | 1.141 |
| 15 | Abengourou | 99766704 | 13.374 | 0.301 | 0.967 | -1.018 | -0.633 | 0.976 | -0.222 | 1.139 |
| 16 | Grand-Bassam | 74825028 | 1.609 | 0.216 | 0.983 | -1.480 | -1.193 | 1.176 | -0.331 | 1.132 |
| 17 | Bongouanou | 74825028 | 21.223 | 0.414 | 0.921 | -0.709 | 0.114 | 0.387 | -0.331 | 0.879 |
| 18 | Arrah | 74825028 | 24.982 | 0.390 | 0.943 | -0.561 | -0.044 | 0.659 | -0.331 | 0.846 |
| 19 | Bondoukou | 74825028 | 18.835 | 0.266 | 0.984 | -0.803 | -0.863 | 1.190 | -0.331 | 0.799 |
| 20 | Séguéla | 49883352 | 43.517 | 0.436 | 0.976 | 0.168 | 0.265 | 1.095 | -0.440 | 0.753 |
| 21 | Dimbokro | 24941676 | 34.444 | 0.384 | 0.976 | -0.189 | -0.084 | 1.086 | -0.549 | 0.642 |
| 22 | Alépé | 74825028 | 22.318 | 0.308 | 0.952 | -0.666 | -0.581 | 0.775 | -0.331 | 0.529 |
| 23 | Soubré | 124708380 | 11.723 | 0.401 | 0.848 | -1.083 | 0.029 | -0.564 | -0.113 | 0.435 |
| 24 | Tanda | 24941676 | 33.360 | 0.344 | 0.960 | -0.232 | -0.344 | 0.883 | -0.549 | 0.222 |
| 25 | Tiassalé | 24941676 | 37.840 | 0.288 | 0.998 | -0.056 | -0.716 | 1.375 | -0.549 | 0.166 |
| 26 | Sinématiali | 49883352 | 27.952 | 0.215 | 0.996 | -0.444 | -1.200 | 1.353 | -0.440 | 0.158 |
| 27 | Gagnoa | 99766704 | 16.351 | 0.374 | 0.862 | -0.901 | -0.146 | -0.381 | -0.222 | 0.152 |
| 28 | M'Batto | 24941676 | 24.560 | 0.375 | 0.902 | -0.578 | -0.138 | 0.137 | -0.549 | 0.028 |
| 29 | Méagui | 99766704 | 10.462 | 0.384 | 0.821 | -1.132 | -0.081 | -0.914 | -0.222 | -0.084 |
| 30 | Agboville | 174591732 | 21.805 | 0.347 | 0.847 | -0.686 | -0.327 | -0.572 | 0.105 | -0.108 |
| 31 | Sikensi | 24941676 | 46.400 | 0.300 | 0.996 | 0.281 | -0.637 | 1.347 | -0.549 | -0.120 |
| 32 | Ferkessédougou | 74825028 | 16.261 | 0.215 | 0.926 | -0.904 | -1.200 | 0.445 | -0.331 | -0.182 |
| 33 | Dabou | 124708380 | 34.483 | 0.237 | 0.951 | -0.188 | -1.052 | 0.766 | -0.113 | -0.211 |
| 34 | Sinfra | 24941676 | 6.867 | 0.403 | 0.805 | -1.274 | 0.045 | -1.117 | -0.549 | -0.347 |
| 35 | Vavoua | 49883352 | 75.297 | 0.527 | 0.940 | 1.417 | 0.867 | 0.627 | -0.440 | -0.363 |
| 36 | Daloa | 74825028 | 23.656 | 0.384 | 0.844 | -0.613 | -0.082 | -0.614 | -0.331 | -0.413 |
| 37 | Mankono | 49883352 | 63.440 | 0.626 | 0.842 | 0.951 | 1.519 | -0.643 | -0.440 | -0.514 |
| 38 | Kounahiri | 24941676 | 80.233 | 0.554 | 0.934 | 1.612 | 1.044 | 0.544 | -0.549 | -0.573 |
| 39 | M'Bahiakro | 24941676 | 57.067 | 0.489 | 0.897 | 0.701 | 0.610 | 0.067 | -0.549 | -0.573 |
| 40 | Lakota | 24941676 | 27.633 | 0.339 | 0.872 | -0.457 | -0.378 | -0.249 | -0.549 | -0.718 |
| 41 | Bocanda | 24941676 | 63.267 | 0.451 | 0.919 | 0.944 | 0.363 | 0.357 | -0.549 | -0.773 |
| 42 | Zuénoula | 124708380 | 65.814 | 0.544 | 0.845 | 1.045 | 0.974 | -0.599 | -0.113 | -0.782 |
| 43 | Bouaflé | 174591732 | 36.493 | 0.469 | 0.777 | -0.108 | 0.480 | -1.481 | 0.105 | -0.787 |
| 44 | Divo | 74825028 | 19.698 | 0.294 | 0.849 | -0.769 | -0.676 | -0.551 | -0.331 | -0.789 |
| 45 | Tengrela | 24941676 | 43.389 | 0.150 | 0.996 | 0.163 | -1.629 | 1.354 | -0.549 | -0.986 |
| 46 | Oumé | 99766704 | 62.546 | 0.417 | 0.890 | 0.916 | 0.135 | -0.023 | -0.222 | -1.026 |
| 47 | Guéyo | 24941676 | 34.800 | 0.387 | 0.817 | -0.175 | -0.060 | -0.961 | -0.549 | -1.395 |
| 48 | Djekanou | 124708380 | 55.199 | 0.384 | 0.846 | 0.627 | -0.080 | -0.582 | -0.113 | -1.402 |
| 49 | Issia | 49883352 | 26.620 | 0.417 | 0.762 | -0.497 | 0.139 | -1.669 | -0.440 | -1.473 |
| 50 | Boundiali | 24941676 | 54.733 | 0.240 | 0.941 | 0.609 | -1.037 | 0.644 | -0.549 | -1.550 |
| 51 | M'Bengué | 24941676 | 43.160 | 0.136 | 0.943 | 0.154 | -1.723 | 0.664 | -0.549 | -1.761 |
| 52 | Jacqueville | 24941676 | 36.900 | 0.235 | 0.860 | -0.093 | -1.068 | -0.405 | -0.549 | -1.930 |
| 53 | Daoukro | 49883352 | 39.944 | 0.427 | 0.732 | 0.027 | 0.205 | -2.054 | -0.440 | -2.316 |
| 54 | Grand-Lahou | 24941676 | 56.667 | 0.229 | 0.871 | 0.685 | -1.110 | -0.263 | -0.549 | -2.606 |
| 55 | Man | 24941676 | 21.640 | 0.295 | 0.710 | -0.693 | -0.668 | -2.337 | -0.549 | -2.861 |
| 56 | Taabo | 24941676 | 124.200 | 0.401 | 0.931 | 3.341 | 0.028 | 0.512 | -0.549 | -3.350 |
| 57 | Bouna | 24941676 | 86.560 | 0.380 | 0.813 | 1.860 | -0.110 | -1.011 | -0.549 | -3.530 |
| 58 | Yakassé- Attobrou | 24941676 | 85.500 | 0.379 | 0.800 | 1.819 | -0.118 | -1.181 | -0.549 | -3.666 |
| 59 | Niakaram -andougou | 24941676 | 45.833 | 0.294 | 0.718 | 0.259 | -0.674 | -2.231 | -0.549 | -3.713 |
| 60 | Touba | 24941676 | 64.800 | 0.301 | 0.761 | 1.005 | -0.628 | -1.687 | -0.549 | -3.869 |
| 61 | Dianra | 24941676 | 116.267 | 0.483 | 0.778 | 3.029 | 0.573 | -1.466 | -0.549 | -4.470 |

**S6 Table. Full list of priority departments.**
