# Supplementary material for: Spatial targeting of Screening + Eave tubes (SET), a house-based malaria control intervention, in Côte d’Ivoire: A geostatistical modelling study
Source: PLOS Glob Public Health. 2021 Nov 15;1(11):e0000030. doi: 10.1371/journal.pgph.0000030 (PMC10021308; doi:10.1371/journal.pgph.0000030)
Supplement: Attachment — Submitted filename: Response to Reviewers.docx [file pgph.0000030.s007.docx]

Dear reviewers,

Thank you for taking the time to review our manuscript. Your comments are very valuables to improve the quality of this work. In this document, we outline the response to your comments, suggestions, and we share some of your concerns and discuss our point of view.

# Reviewer #1

General comments:

**In this study, the authors used a geostatistical model to predict household suitability for Screening + Eave Tubes (SET) in Cote d'Ivoire (defined based on housing materials), and then used the model output alongside other factors to identify priority areas for ground-truthing for potential SET implementation. The study was based on 2011 DHS data from Cote d’Ivoire, which are the latest publicly available national-level georeferenced data. This study addresses the important issue of local adaptation and targeting of interventions for malaria control and elimination, and it provides a data-driven approach to identifying locations that could be prioritized for implementation of a promising house-based intervention. It also provides estimates of cost and highlights the need for financing strategies to bring SET interventions to scale. The article is well-written and methods are well explained, however some aspects could be further clarified.**

We acknowledge the comment made here as it shows the main idea of the manuscript was passed as we wanted. We also appreciate the positive comments and the highlights of this work. We try to clarify some of the aspects that were mentioned.

Specific comments:

**1. A more informative title with location and study type would be useful; E.g. “Spatial targeting of Screening + Eave Tubes (SET), a house-based malaria control intervention, in Cote d’Ivoire: a geostatistical modelling study”**

We have changed the title. We agreed that our title was too broad. Although done in Cote d’Ivoire, this intervention is applicable in other contexts, specifically in sub-Saharan Africa.

**2. Abstract: the objective statement indicates the study aims to “define the most appropriate locations where Screening + Eave Tubes (SET) could be implemented”, but this is a bit misleading since the intent is not to directly inform intervention implementation, rather it is a proof of principle study to generate estimates that would require further validation prior to any implementation. Suggest modifying the abstract to match the objective statement in the introduction, i.e. “identify priority areas for ground-truthing of geostatistical model output”.**

We agreed about the misleading use of the word implemented. We mentioned during the manuscript that the geostatistical output needs ground-truthing. Therefore, we have changed the aim to “predict household suitability and define the most appropriate locations for ground-truthing where Screening + Eave Tubes (SET) could be implemented across Côte d’Ivoire”.

**3. In the abstract, more information is needed on how “suitability of SET” and “impact” are defined.**

Thank you for this suggestion. We have added a sentence to define the suitability of SET by stating that suitability was based on the household wall and roof materials. We substituted the word "impact", for “potential population coverage and cost”, which was how we were assessing the potential impact of SET.

**4. Introduction: here it is apparent that suitability refers to the presence of improved house construction, but “impact” is not described in the introduction and should be described here if it is an objective.**

Aligned with the point raised above, we have added to the aim “calculate the potential population coverage and cost” as a means of defining impact.

**5. Methods, line 77: Some rationale for using DHS data from 2011 should be presented in the methods, considering that the information is ten years old, and does not accurately reflect the current status of housing materials in many areas. Indeed, the introduction mentions that “in the past decade, there has been an intense urbanization and house improvement in sub-Saharan Africa”. Did the authors request GPS data from the 2016 MICS a limitation of the study that affects all estimates derived from the model output, therefore a more cautious tone would be appropriate to frame the study as a proof of principle (see comment #8 and #10 below).**

We tried to get the 2016 MICS geospatial dataset, but we could not obtain it by the absence of a response from the people of the national statistics department of the Ivory Coast. We have addressed comments #8 and #10 to provide support to the 2011 DHS dataset.

**6. Methods, line 79-80: it would be useful to define “appropriate” in the body of the paper, especially since this definition essentially delineates between urban and rural houses (as mentioned in the results lines 157-159).**

We have added the definition of appropriate materials into the body of the paper.

**7. Methods: the authors describe a sequence of data processing steps that they followed to calculate the target population and cost, based on the gridded model output. They then derive department-level variables from the model output (exceedance probability and included area) and combine these with department-level data on malaria parasite rate and travel time to produce a ranking of departments to prioritize for ground-truthing based on summed z-scores. The approach that was used appears sound, however there is a high likelihood of introducing error at each step, making the end results less robust. A figure showing the data processing steps (e.g. flow chart) would be useful so that it is clear which data were aggregated or combined for which outcome measure.**

We recognise the risk about intrinsic errors of adding different layers of information each of them calculated independently. However, it was the rank and not the final value that was of interest, therefore, compounding errors have less impact to the ranking estimates in this particular point as are all added to the departments in the same measure.

We have added a flow chart (Fig 2) describing each process, so the reader has a clear understanding.

**8. Methods: Did the authors consider conducting a sensitivity analysis to see what changes would result from variability in the model input measures – for example, if it was assumed that 20% (or perhaps more, depending on the rate of urbanization) of houses in each cluster were improved between 2011 and 2021 how would this affect the estimates? Presumably more areas would meet the exceedance threshold than were identified by the initial model.**

We have done a sensitivity analysis as suggested. We used the 20% change over time as we used as reference the work done by Tusting et al. 2019. Although the parameters they use are not just wall and roof materials but improved houses, they estimated an improvement on the household characteristics by roughly 20%.

We re-ran the geostatistical model and then we filtered the results to get priority ground-truthing areas and to calculate the potential coverage. We have added the sensitivity analysis as a supplementary file 6. Main changes in the manuscript are in the methodology (125 – 129) and discussion (257 – 262).

**9. Results, line 216-218: is there a word missing between ‘theoretically’ and ‘SET’?**

There was indeed missing a word. Wording was changed here.

**10. Discussion: the limitations of the 2011 DHS data are explicitly mentioned, but it is not clear what is meant by “To account for the out-to-date housing data and the influence of the covariates in the model, we created an operational scenario to help guide and target the implementation or future trials”.**

The authors agreed the sentence was not clear and did not add much to the whole discussion of data limitation. Therefore, we decided to remove it from the discussion as the ground-truthing outcome and the covariates used in the model were discussed in other paragraphs.

# Reviewer #2:

General comments:

**SET has demonstrated substantial public health benefit in a randomised control trial in Cote d’Ivoire though it is unclear where it can be implemented given the requirement of concrete housing. This manuscript sets out a geostatistical method of assessing where it might be useful to introduce and estimates the cost. There is a need for this type of work and the manuscript uses sensible geospatial methods. The paper is well written and is generally well written, though I fear the resolution of these data and the ecological fallacy makes me question some of the results.**

We thank the feedback on this work. We acknowledge that the data resolution is not the most appropriate for the type of work we did. On one hand, there is not currently a better publicly available dataset, nor in granularity, spatial or temporal resolution. On the other hand, this work is intended as a proof of concept for a new vector control intervention to provide insights into feasibility, scalability and ground-truthing.

Specific comments:

**1). The major problem as I see it is one of geographical scale. You are looking at an intervention which requires concrete housing which are more likely in urban areas. It is not mentioned in the manuscript but it is widely understood thar malaria is less common in heavily urbanised area due to the lack of availability of suitable mosquito breeding sites. Therefore areas most suitable to SET might have the least need for it from a malaria control perspective. Malaria data is taken from DHS data summaries at a very high geographical scale (admin1) and as i understand it DHS is generally collected in rural areas. Therefore the suitability of the admin 3 is driven by this crude approximation of whether the location is of moderate or high transmission. In the crudest scenario you might have an admin 3 unit with a large urban area with all concrete houses but no malaria and a small rural area with lots of malaria but no concrete houses. The site of the CRTC was clearly high malaria and widespread concrete housing but this is unlikely to be universal, suggesting the numbers who could be protected and the costs could be exaggerated. This does not invalidate the models identifying areas with concrete housing, but the authors might want to consider using more fine resolution outputs from the MAP to identify high malaria areas or perhaps using maps of rural/urban breakdown to exclude those urban areas in a sensitivity analyses. Either way the issue needs to be highlighted.**

The malaria data that were used in our analysis came from the MAP at a spatial resolution of 5km, not the DHS dataset as indicated by the reviewer. Information on the covariates of the model (parasite rate included), the covariate data, and the data sources are specified in the methods and supplementary material (Figure S3 and Table S3). The same data was used to characterise areas as low/moderate/high transmission in the ranking exercise. We believe this should address the reviewer’s concern.

**2). Title seems inappropriate. It could be argued that LLINs and IRS are house-based malaria control and certainly there are other housing improvements that could be implemented in houses unsuitable for SET. Consider revising.**

We have changed the title. We agreed that our title was too broad. Although this was done in Cote d’Ivoire, this is an intervention applicable in other contexts, specifically in sub-Saharan Africa.

**3). Line 21 of the abstract says that the work “and to calculate the impact” of SET, but this does not appear to be the case.**

Indeed, the use of the word impact could be misleading or misinformative, therefore we decided to change to calculate the coverage and cost as it was what we meant by impact. We altered this also in the main aim in the manuscript body.

Minor points

**1). Language needs to be tighter. In the abstract as it says “SET could protect 31% of the total population and 17·5% of the population in areas of high malaria transmission”. As I understand it 31% of structures people live in the could implement SET. Other people in these communities could benefit from SET (as is mentioned) so saying just those using it are protected is a little misleading. Similarly, you are talking about high transmission admin 1 units (districts?) and this should be stated here as there will be other high transmission areas within low transmission districts.**

31% percent represents the population living in areas which were deemed suitable for SET. The wording has been changed to make this clearer. As indicated in a previous response, characterisation of transmission was done at a spatial resolution of 5km, not at the admin 1 level.

**2). Latin names of mosquitoes should be written in full the first time they are used (even if the genus has been abbreviated before, eg. Line 70).**

Agreed, changed.

**3). Line 117 says that “The trial results [16] also suggest community protection is achieved when ≥70% of households have SET”. This indicated that community protection is either on or off, when it has been established that it is more of a continuum. Consider changing.**

Good point. We changed the text by providing more information from the trial paper.

**4). Line 208 typo “Supporting the either….”**

Agreed, removed “the”.
